# Role of type I interferons in inflammasome activation, cell death, and disease during microbial infection

**DOI:** 10.3389/fcimb.2013.00077

**Published:** 2013-11-12

**Authors:** R. K. Subbarao Malireddi, Thirumala-Devi Kanneganti

**Affiliations:** Department of Immunology, St. Jude Children's Research HospitalMemphis, TN, USA

**Keywords:** type I IFN, IFNβ, NLR, caspase-1, inflammasome, cell death, Nlrp3

## Abstract

Interferons (IFNs) were discovered over a half-century ago as antiviral factors. The role of type I IFNs has been studied in the pathogenesis of both acute and chronic microbial infections. Deregulated type I IFN production results in a damaging cascade of cell death, inflammation, and immunological host responses that can lead to tissue injury and disease progression. Here, we summarize the role of type I IFNs in the regulation of cell death and disease during different microbial infections, ranging from viruses and bacteria to fungal pathogens. Understanding the specific mechanisms driving type I IFN-mediated cell death and disease could aid in the development of targeted therapies.

## Introduction

**Figure 1:**
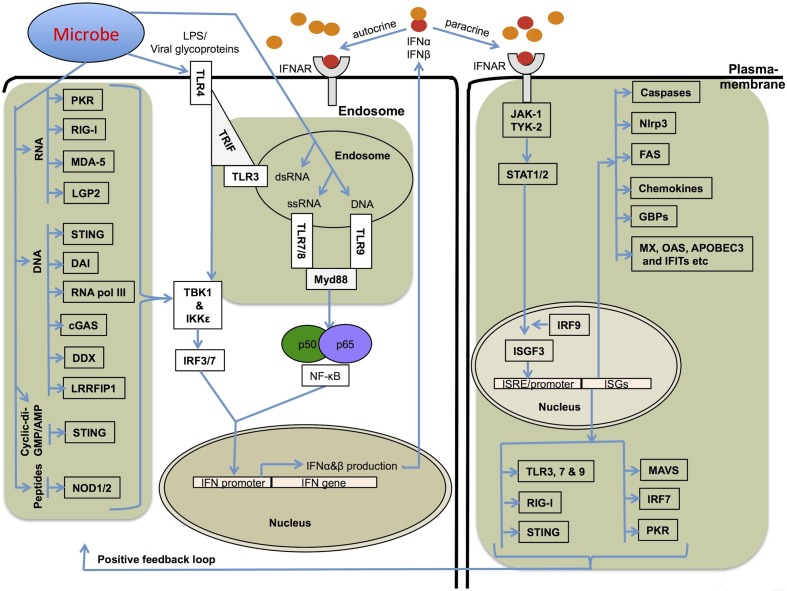
**Mechanisms involved in type I IFN production and its receptor signaling**. Several different stimuli including pathogen derived LPS, glycoproteins, RNA and DNA can induce type I IFNs (IFNα and IFNβ) via upstream pattern recognition receptors. TLRs sense the endosomally located PAMPs (pathogen associated molecular patterns) and DAMPs (damage associated molecular patterns) and recruit TRIF (TRL3 and TLR4) or Myd88 (TLR4, 7, 8, and 9), which further transfer the signals downstream to IRF3 and IRF7. RLRs, NLRs, DAI, STING, and related receptors sense nucleic acids in the cytoplasm. The RNA sensors, RIG-I, MDA-5, and LGP2, and DNA sensor STING use the adapter protein MAVS (mitochondrial antiviral signaling protein) to transfer signals to IRFs for type I IFN transcription. RIG-I and MDA-5 also sense ligands generated by RNA polymerase III from cytoplasmic DNA PAMPs. All these pathways utilize the common downstream kinases, TBK1/IKKε for activating Interferon transcription factors. Type I IFNs bind to IFNAR receptor and activate a robust transcriptional pathway through a JAK-STAT signaling pathway. The transcriptional complexes activated by type I IFN signaling bind to specific ISRE/GAS sequences and lead to the expression of several genes important for cell death, cell proliferation and immune responses.

Interferons (IFNs) are broadly classified into three groups, which are denoted as type I, II, and III based on the specific receptor utilization for their signal transduction. The type I IFN family comprises subtypes of IFNα (13 subtypes), IFNβ, IFNω, and IFNε (Pestka et al., 2004; Hertzog and Williams, 2013). All of the type I IFNs bind to a common heterodimeric receptor, called the IFNα/β receptor (IFNAR), composed of two chains, IFNAR1 and IFNAR2, that are associated with the tyrosine kinases Tyk2 and Jak1. Activated Tyk2 and Jak1 recruit and phosphorylate several signal transducer and activator of transcription (STAT) family members (Figure 1) (Pestka et al., 2004; Platanias, 2005). Activated STAT1 forms a dimer with STAT2, leading to the recruitment of IRF9 and subsequent formation of a heterotrimeric complex called IFN-stimulated gene factor 3 (ISGF3) (Figure 1). This complex translocates to the nucleus, where it binds upstream IFN-stimulated response elements (ISRE) and activates the transcription of type I IFN-inducible genes (Pestka et al., 2004; Platanias, 2005). Type I IFNs are classically known for their antiviral immune responses; however, several studies have demonstrated that a wide range of non-viral pathogens can also induce their expression. However, the specific mechanisms and physiological consequences of IFN responses to such pathogens are poorly understood. Various studies have attributed contrasting roles and differential outcomes to type I IFNs in immune responses to diverse microbial pathogens. The ability of IFNs to regulate cell death has been known for a long time and recent studies have started to reveal the specific mechanisms involved.

During the course of evolution, the arms race between bacterial pathogens and host organisms has resulted in the development of virulence mechanisms by microbes and the reciprocal development of host counter strategies to efficiently defend against them. Cell death has emerged as one of the important aspects of such a race between microbes and the host, exploited by both, while the final outcome is dependent on the specific pathogen and cell types involved. The in depth molecular mechanisms of how type I IFN signaling causes differential outcomes during different microbial infections remains to be established. Here we discuss the role of type I IFNs in regulating cell death and disease in various infection models and highlight its emerging role in inflammasome activation.

## Intracellular bacteria

### listeria

*Listeria monocytogenes* (LM), a gram-positive intracellular bacterium that grows rapidly inside host cells, is known to potently induce type I IFN production in mouse (Havell, 1986; O'Riordan et al., 2002) and human (Reimer et al., 2007) macrophages. LM causes life-threatening infections in immunocompromised individuals and may lead to septic abortion in pregnant women (Pamer, 2004). Upon internalization by phagocytes, LM escapes the early phagosome by secreting a hemolytic toxin, Listeriolysin O (LLO) (Portnoy et al., 1988). *Listeria* mutants that do not express the LLO toxin fail to escape the phagosome or to induce IFN-β production. LM-induced type I IFN production is largely independent of TLR signaling and is instead mediated by cytoplasmic RLR- and STING-dependent pathways through the TBK1-IRF3 signaling axis (Ishikawa et al., 2009; Woodward et al., 2010). In addition, *in vivo* studies demonstrated that *Irf*3^−/−^ and *Ifnar*^−/−^ mice, which neither make nor respond to type I IFNs, respectively, are highly resistant to *L. monocytogenes* infection (Auerbuch et al., 2004; Carrero et al., 2004; O'Connell et al., 2004). Furthermore, priming with poly(I:C), a well known type I-inducing agent, results in enhanced death rate in LM-infected WT mice but not in *Ifnar*^−/−^ mice (O'Connell et al., 2004), indicating a detrimental role for type I IFNs during *Listeria* infection. Moreover, LM-infected *Ifnar*^−/−^ mice were shown to have reduced lymphocyte and splenocyte apoptosis and antibody-dependent neutralization of LLO controlled infection that resulted in reduced pathology (Auerbuch et al., 2004; Carrero et al., 2004; O'Connell et al., 2004). However, Rayamajhi et al. have proposed an alternative mechanism, where type I IFNs-dependent down-regulation of IFNγ receptor results in heightened susceptibility of wild type mice to *Listeria* infection (Rayamajhi et al., 2010).

*Listeria* is known to trigger assembly of multiple types of inflammasomes that include Aim2, Nlrc4, and Nlrp3 for caspase-1 activation in mouse macrophages. During *Listeria* infection, type I IFNs have significant roles in regulation of inflammasome activation and pyroptosis (Henry et al., 2007; Kim et al., 2010; Rathinam et al., 2010; Wu et al., 2010). A few studies have attempted to dissect the relative contributions of each of these inflammasomes and demonstrated that the detection of DNA by AIM2 receptor was indispensable for inflammasome activation and pyroptosis during *Listeria* infection in human PBMCs and mouse macrophages (Kim et al., 2010; Rathinam et al., 2010; Sauer et al., 2010). In addition, Sauer et al. demonstrated that pyroptosis was totally dependent on Aim2, while the Nlrp3 and Nlrc4 inflammasomes were dispensable for this process (Sauer et al., 2010). These reports implicate that type I IFNs play a role in the efficient induction of inflammasome activation and pyroptosis.

### legionella

*Legionella pneumophila* is an intracellular, gram-negative bacterial pathogen that replicates in host macrophages and causes a severe pneumonia called Legionnaires' disease. Lipmann et al. reported that *L. pneumophila*-infected mouse macrophages produce IFNβ in a STING- and IRF3-dependent manner (Stetson and Medzhitov, 2006a; Lippmann et al., 2011). By contrast, Monroe et al. have demonstrated that *L. pneumophila* RNA also stimulated a Rig-I-dependent IFN response and proposed that *L. pneumophila* RNA, or host RNA, rather than *L. pneumophila* DNA, as the primary ligand that stimulates the host IFN response (Monroe et al., 2009). IFN-αβ inhibits *L. pneumophila* replication in the permissive A/J or CD1 mouse macrophages (Schiavoni et al., 2004). Furthermore, Bastian et al. have reported that *Legionella* is controlled by IFNβ induced in human lung epithelial cells via MAVS and IRF3 (Opitz et al., 2006). Dendritic cells (DCs) and macrophages are capable of restricting *L. pneumophila* growth through NAIP5-dependent caspase-1 activation and cell death. However, DCs were shown to undergo a more rapid apoptosis than macrophages, leading to enhanced restriction of *Legionella* growth (Nogueira et al., 2009). Indeed, eliminating the pro-apoptotic proteins BAX and BAK or over-expressing the anti-apoptotic protein BCL-2 were both found to restore *L. pneumophila* replication in DCs (Nogueira et al., 2009). Furthermore, a sub-population of DCs, plasmacytoid DCs (pDCs) is known to express higher levels of IFNs (Liu, 2005), which can potentially contribute to the higher cell death responses to *Legionella*. How *Legionella*-induced type I IFN promotes cell death is not well understood currently, however, one possible mechanism might be that Type I IFN-dependent up regulation of pro-cell death molecules like BAK and TRAIL can potentiate apoptosis (Fuertes Marraco et al., 2011; Cohen and Prince, 2013).

### mycobacterium

*Mycobacterium* spp. are pathogenic intracellular bacteria that cause tuberculosis (TB) and leprosy. Human and mouse myeloid cells secrete type I IFNs in response to mycobacterial infections (Pandey et al., 2009; Berry et al., 2010; Novikov et al., 2011). Blood based profiling has identified type I IFN-induced genes as the most striking characteristic signature of active TB (Berry et al., 2010). In addition, Wu et al. have reported that several TB-induced genes have key transcription factor binding sites for STATs, IRF-1, IRF-7, and OCT-1 (Wu et al., 2012). IFN-β and its downstream genes, including interleukin-10 (IL-10), were induced in monocytes by *M. leprae in vitro* and were preferentially expressed in progressive lepromatous lesions (Teles et al., 2013). Manca et al. have reported that type I IFNs enhance the virulence of *M. tuberculosis* by suppression of Th1 type immune responses. They have also shown that treatment with purified IFN-α/β increases lung bacterial loads, resulting in reduced survival in mice (Manca et al., 2001). In addition, another study reported that treatment with exogenous type I IFN results in a striking loss of mycobacteriostatic activity in monocytes and macrophages (Bouchonnet et al., 2002). Furthermore, Mayer-Barber et al. have shown that both IL-1α and IL-1β are critical for host resistance to TB and provided evidence that type I IFNs suppressed IL-1 production (Mayer-Barber et al., 2011). However, early clinical studies suggested that type I IFNs have beneficial effects against pulmonary TB (Giosue et al., 1998; Palmero et al., 1999). Together, these studies indicate that the role of type I IFNs in mycobacterial infections is debatable and requires further research to establish a clear consensus.

### francisella

*Francisella tularensis* is a gram-negative bacterium and causes tularemia. *F. tularensis* is classified as a Class A agent (having a high potential for use as a bioweapon) by United States regulators, due to its high virulence and ability to spread rapidly. Cytosolic recognition of *Francisella* induces type I IFN production in an IRF3-dependent manner (Henry et al., 2007). *Francisella* induces Aim2 inflammasome-dependent pyroptosis, which further depends on the ability of the bacterium to induce type I IFN production (Henry et al., 2007; Fernandes-Alnemri et al., 2010). Consistent with this, *Francisella*-induced Aim2 inflammasome activation and IL-1β secretion are abrogated in macrophages derived from bone marrow of *Irf3*- and *Ifnar*-deficient mice (Fernandes-Alnemri et al., 2010; Jones et al., 2010; Rathinam et al., 2010). Wild-type *Francisella* that can escape into the cytosol induces type I IFN and Aim2 inflammasome activation and host cell death (Mariathasan et al., 2005; Henry et al., 2007; Jones et al., 2010). This observation underscores the importance of cytosolic recognition of bacteria or their components (DNA in the case of *Francisella*) as an important innate immune mechanism to trigger inflammasome activation. Although studies in mice certainly support an important role for Aim2 in immune responses to *Francisella*, its role in human monocytes seems to be less prominent due to its lack of expression and/or induction in response to type I IFNs (Gavrilin and Wewers, 2011).

### salmonella

*Salmonella enterica* serovar Typhimurium (*S. Typhimurium*) is a gram-negative, intracellular pathogen that is quickly cleared by macrophages. This pathogen is a leading cause of acute gastroenteritis worldwide, which is transmitted primarily via the consumption of contaminated food or water. Induction of rapid cell death is a virulence strategy for this pathogen and contributes to dampening host innate immune responses (Lindgren et al., 1996). Robinson et al. have reported that *Salmonella* exploits type I IFN signaling for eliminating macrophages to establish infection (Robinson et al., 2012). Their studies also revealed that type I IFN-induced cell death of the macrophages is mediated by the classical RIP1-RIP3 dependent necroptosis pathway (Robinson et al., 2012). *Salmonella* is detected by NLRP3 and NLRC4 inflammasomes resulting in casapse-1 activation and pyroptosis (Franchi et al., 2006; Miao et al., 2006; Broz et al., 2010). Broz et al. have suggested the existence of an IFN-inducible regulator of caspase-11 that is crucial for activation of non-canonical Nlrp3 inflammasome activation in mutant *Salmonella-* [type 3 secretion system mutant, i.e., *Salmonella* pathogenicity island 1(SPI-1)] infected mouse macrophages (Broz et al., 2012). Their study revealed that *Salmonella* induces expression and activation of caspase-11 through a Toll-like receptor 4 (TLR4)-dependent and TIR-domain containing adaptor-inducing IFN-β (TRIF)-mediated IFNβ signaling pathway. Consistent with this, *Ifnar1*^−/−^ or *Irf3*^−/−^, or *Stat-1*^−/−^ macrophages infected with mutant *Salmonella* did not process the casapase-11 or activate the non-canonical cell death pathway. Furthermore, *in vivo Casp1*^−/−^ mice are more susceptible to Salmonella infection than the *Casp1*^−/−^ *Casp11*^−/−^ mice (Broz et al., 2012). Thus, these results indicate that caspase-11 mediated cell death results in detrimental effects to the host. Together, it is evident that *Salmonella* exploits type I IFN signaling to rapidly kill the immune cells to cause disease in the host.

## Extracellular bacteria

### staphylococcus

Staphylococcal infections have recently emerged as a significant problem to human health, due to the emergence of antibiotic resistant strains that cause life-threatening infections, especially in post-influenza exposures (Klevens et al., 2007; Martin et al., 2009; David and Daum, 2010). *S. aureus* infected mouse and human epithelial cells produce type I IFN in a STAT3-dependent manner in response to its virulent protein A (Martin et al., 2009). In mouse myeloid DCs and macrophages, TLR9 and IRF1 have important roles (Schmitz et al., 2007), while the cell wall component lipoteichoic acid (LTA) utilizes the IRF1-STAT1 axis in mouse macrophages to induce type I IFNs (Liljeroos et al., 2008). Absence of IFNAR signaling results in protection against lethal *S. aureus* pneumonia infection compared to wild-type control mice (Martin et al., 2009). Following recognition of *S. aureus* α-hemolysin, mouse macrophages undergo pyroptosis in an Nlrp3 inflammasome-dependent manner (Mariathasan et al., 2006; Craven et al., 2009). In contrast to Martin et al., a recent report demonstrated that IFNα induces phospholipid scramblase 1 (PLSCR1) in human lung epithelial cells as part of an innate protective mechanism to a bacterial pore-forming toxin (Lizak and Yarovinsky, 2012) and another study demonstrated a protective role of CpG DNA (a potent inducer of type I IFN production) in a mouse model of *S. aureus* pneumonia (Roquilly et al., 2010). Furthermore, Kaplan et al. have shown that phagosomal degradation and cytosolic release of intracellular ligands are essential for the induction of IFN-β in mouse and human DCs, which is required for the host defense against *S. aureus* during cutaneous infection in mice (Kaplan et al., 2012). Taken together, these reports suggest that type I IFNs can have both protective and detrimental roles during *S. aureus* infection. The disease outcome is variable and may depend on the immune status of the host, the site of infection and the specific strains causing the infection.

### streptococcus

*S. pneumonia* causes acute lung infections and activates type I IFN expression (Joyce et al., 2009; Parker et al., 2011). DAI (DNA-dependent activator of IFN-regulatory factors) dependent recognition of bacterial DNA is proposed to be responsible for inducing type I IFN expression through cytoplasmic DNA sensing pathway involving STING, TBK1 and IRF3-dependent signaling pathways (Parker et al., 2011). Type I IFN treatment enhances protection of mice against *S. pneumoniae* (Weigent et al., 1986). However, prior exposure to influenza A virus leads to increased susceptibility to bacterial infections in a type I IFN-dependent manner (Morens et al., 2008; Shahangian et al., 2009). As opposed to bacterial infection alone, type I IFNs produced during secondary infection with *S. pneumonia* inhibits production of chemokines like CXCL1 and CXCL2 and sensitizes hosts to secondary bacterial pneumonia (Shahangian et al., 2009).

### pseudomonas

*Pseudomonas aeruginosa* is a causative agent of pneumonia and infection in cystic fibrosis (CF) patients is associated with significant mortality (Zhuo et al., 2008). *P. aeruginosa* induces type I IFN expression predominantly through the TLR4-TRIF-IRF3 axis (Parker et al., 2012). TLR4 signaling was shown to be important for clearance of *P. aeruginosa* from the lungs and preventing sepsis in infected hosts (Faure et al., 2004; Ramphal et al., 2005; Skerrett et al., 2007; Cohen and Prince, 2013). Similarly, *Trif* and *Irf*3 deficiencies in mice resulted in reduced expression of type I IFN-induced chemokines including CXCL10 (IP-10) and CCL5 (RANTES) and abrogated neutrophil recruitment to the lungs leading to impaired bacterial clearance (Power et al., 2007; Carrigan et al., 2010). These results indicate a protective role for type I IFNs during *P. aeruginosa* infection (Roy et al., 2013). *P. aeruginosa* infection of mouse macrophages activates the Nlrc4 inflammasome and induces pyroptosis in a flagellin independent manner (Sutterwala et al., 2007). However, later studies demonstrated a requirement of cytosolic flagellin for Nlrc4 inflammasome activation (Miao et al., 2008; Arlehamn and Evans, 2011). Bacterial expression of specific adhesins, lipopolysaccharide, and a functional type III secretion system were all shown to be necessary to evoke apoptosis (Sutterwala et al., 2007) and the cytotoxin, ExoU-expressing *P. aeruginosa* strain has been shown to inhibit caspase-1 dependent pyroptosis (Sutterwala et al., 2007).

### anthrax

*Bacillus anthracis* is a gram-positive, aerobic bacterium that causes severe pulmonary, gastrointestinal, and cutaneous infections (Dixon et al., 1999). Production of the lethal toxin (LeTx) by this bacterium causes extensive cell death, tissue damage and systemic disease. LeTx is composed of a protective antigen (PA) and lethal factor (LF). Gold et al. found that endogenous IFNs (type I and II) inhibit the germination of *B. anthracis* spores, but exogenous application enhanced inflammation thereby increasing mortality (Gold et al., 2007). In addition, Walberg et al. showed that recombinant murine IFNβ or type I IFN inducers like poly(I:C) provide marked protection against “Inhalation Anthrax” (Walberg et al., 2008). *B. anthracis* LeTxs activate the Nlrp1b inflammasome and pyroptosis in mice (Boyden and Dietrich, 2006; Bergsbaken et al., 2009). It is recognized that macrophages from inbred mice may or may not be sensitive to *B. anthracis-*induced pyroptosis based on the presence of the Nlrp1b inflammasome responsive *Nlrp1b^S/S^* or non-responsive *Nlrp1b^R/R^* alleles (Moayeri et al., 2010; Terra et al., 2010). In this case, pyroptosis of macrophages is believed to counter anthrax infection since IL-1β released during this process helps to generate protective neutrophil responses. It will be essential to study the contribution of type I IFN in pyroptosis induction during *B. anthracis* infection and investigate whether type I IFN can promote apoptosis of neutrophils and initiate other possible detrimental effects.

## Viruses

IFNs were originally discovered as antiviral molecules. Viruses are considerably smaller than other microbial pathogens but represent a major threat to human and other animal health. Extensive progress has been made in understanding the mechanisms of type I IFN production in response to viruses (Stetson and Medzhitov, 2006b; Gonzalez-Navajas et al., 2012; MacMicking, 2012). The diverse mechanisms of cellular entry and tropism of viruses are detected by different TLRs that are strategically located in different cellular compartments (Figure 1). Endosomally-located PRRs including TLR3, 7, 8, and 9 are known to trigger type I IFNs (Figure 1). TLR7 and TLR9 have particularly important roles in plasmacytoid DCs. Once the virus enters the host cytoplasm, several cytoplasmic receptors such as RIG-I-like receptors (RLRs RIG-I, MDA5 and LGP2) and NOD-like receptors (NOD1 and NOD2) monitor the cytoplasm for microbial PAMPs and initiate type I IFN production and associated immune responses (Figure 1).

Programmed cell death is a critical host defense against viruses and type I IFNs are known to be involved to this process. Multiple viruses have been discovered to encode proteins that function to subvert host-induced cell death during infection (Bowie and Unterholzner, 2008; Galluzzi et al., 2010; Kaminskyy and Zhivotovsky, 2010; Gregory et al., 2011). Death of the infected cells is detrimental to viral replication and amplification of viral progeny. However, death of the infected cells can also facilitate viral egress and enhance pathogenesis. Therefore, different viruses have evolved complex mechanisms to enhance or inhibit different forms of cell death (Kaminskyy and Zhivotovsky, 2010). Microarray based studies revealed that a large number of genes are regulated by type I IFNs and several of them are involved in cell death (Der et al., 1998; de Veer et al., 2001; Hertzog and Williams, 2013; Rusinova et al., 2013). However, the mechanisms driving cell death that involve proteins encoded by many of these genes are still awaited.

Uncontrolled chronic viral infections can result in sustained expression of type I IFNs with detrimental pathophysiological outcomes. Two recent studies reported the role of type I IFNs in viral persistence during lymphocytic choriomeningitis virus (LCMV) infection (Teijaro et al., 2013; Wilson et al., 2013). Results from these reports show that robust and acute type I IFNs secreted during early in the infection serve to control viral replication and spread by promoting apoptosis of infected cells and enhancing T cell activation. During chronic infection, prolonged expression and exposure to type I IFNs leads to lymphocyte exhaustion, and reduced viral clearance due to the presence of increased immunosuppressive molecules like IL-10 and PD-L1. Loss of circulating pDCs has been documented in chronic viral infections in mice and humans, which correlates with uncontrolled viral loads, reduced T cell counts and onset of opportunistic infections (Swiecki et al., 2011). However, the mechanism of type I IFN-dependent pDC apoptosis is not entirely clear and represents an important subject for future research.

Type I IFNs promote cell death in multiple ways. Balachandran et al. have demonstrated that type I IFN and protein kinase R (PKR) can sensitize cells to apoptosis primarily through the FADD/caspase-8 pathway (Balachandran et al., 2000). In this study, stimulation of mouse cells with IFN-α/β resulted in enhanced apoptosis and reduced viral replication. In a follow up study, Ezelle et al. showed that the poxvirus-encoded protein CrmA was able to inhibit both viral infection- and dsRNA-mediated apoptosis (Ezelle et al., 2001). Both HSV and vaccinia virus affect PKR and RNase L-mediated apoptosis pathways that are activated by dsRNA products released during viral replication (Der et al., 1997; Diaz-Guerra et al., 1997; Kibler et al., 1997). Influenza viruses cause severe lung pathology leading to lung failure and mortality. Type I IFNs have been recognized to mediate induction of pro-apoptotic TRAIL leading to excessive cell death and tissue injury (Hogner et al., 2013). Infection or treatment with type I IFN induces pro-apoptotic genes; IFN-stimulated gene 54 (ISG54) or IFN-induced gene with tetratricopeptide repeats 2 (IFIT2) that promotes apoptosis by mitochondrial-associated BCL2 family proteins (Reich, 2013).

## Fungi

While type I IFNs are widely known as anti-viral factors, which are either protective or detrimental in bacterial infections, their role in fungal infections is poorly defined. Recently, two reports have shown that *Candida* spp. induce IFN-β in mouse bone marrow-derived DCs (BMDCs) and macrophages (Biondo et al., 2008, 2012; Bourgeois et al., 2011). Different forms of fungal glucans and mannans are sensed by a wide range of innate pattern receptors like TLRs, CLRs, dectins, and mannose receptors and initiate MyD88–mediated NF-κB and MAPK pathways or SYK-CARD9 signaling for cytokine induction and cell death (Netea et al., 2008; Brown, 2011). During fungal infections, type I IFNs are also produced by a TLR-independent pathway requiring RNA sensor MAVS and IRF3 (Inglis et al., 2010). In contrast, Del Fresno et al. have recently reported that *Candida albicans* induces type I IFN in DCs through a DECTIN-1, SYK-, and CARD9-dependent pathway that requires IRF5-mediated transcription but not IRF3 or IRF7 (Del Fresno et al., 2013). Jensen et al. have reported that poly(I:C)-induced or exogenously added IFNα and IFNβ treatments of macrophages suppress anti-*Candida* immune responses and cause death of infected mice (Worthington and Hasenclever, 1972; Jensen et al., 1992; Jensen and Balish, 1993). In addition, *Ifnar*^−/−^ mice are extremely resistant to otherwise lethal *Candida* and *Histoplasma* infections (Inglis et al., 2010; Majer et al., 2012). These studies demonstrated that type I IFNs induce severe kidney damage by promoting excessive recruitment and activation of inflammatory monocytes and neutrophils. However, other reports suggest that type I IFN can be beneficial as part of the host immune response to *C. albicans* (Biondo et al., 2011; Del Fresno et al., 2013).

## Role of interferons in inflammasome activation and pyroptosis

**Figure 2:**
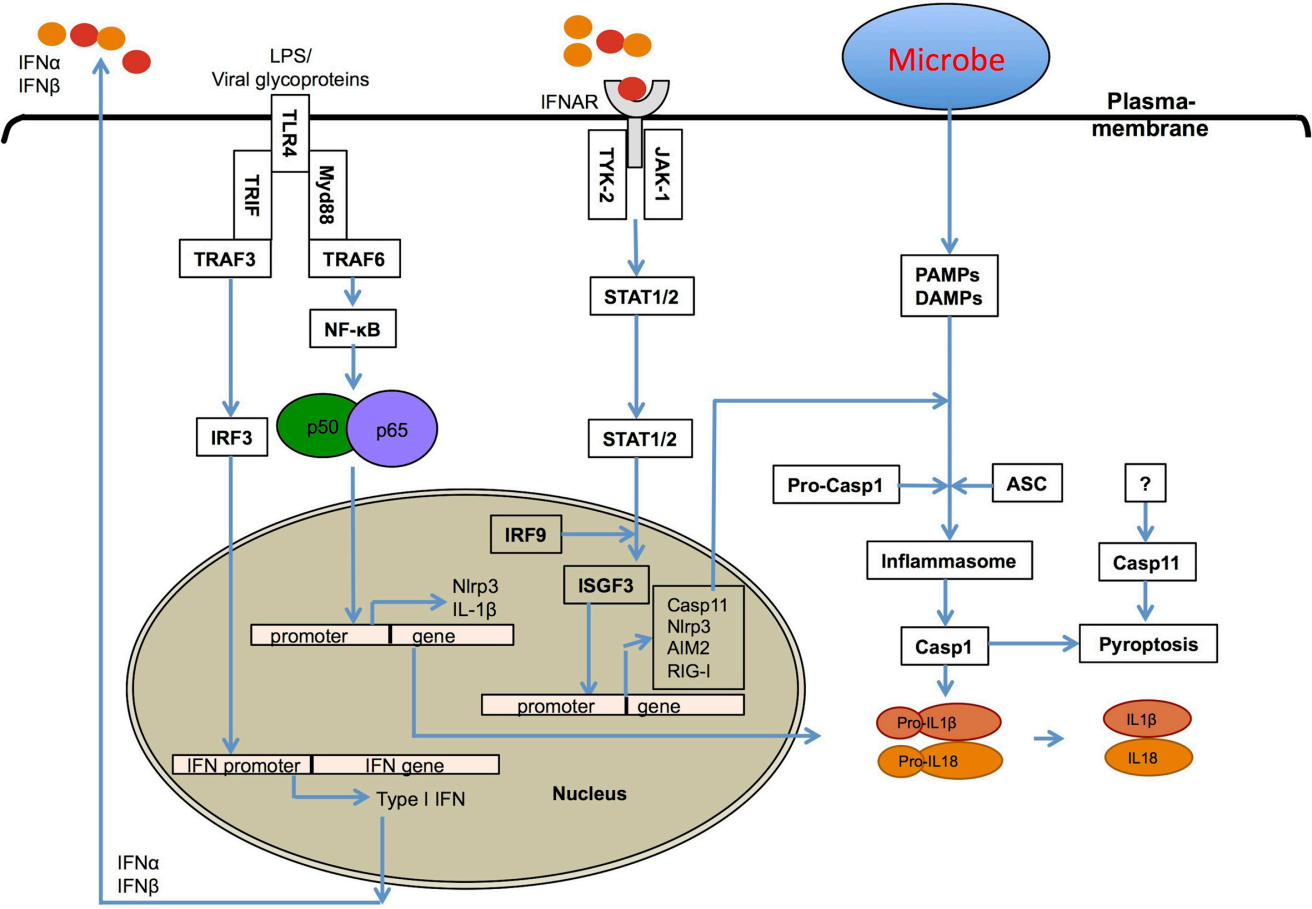
**Role of type I interferons in inflammasome activation**. Type I IFNs contribute to inflammasome activation through two different mechanisms. First, type I interferons are required for the upregulation of caspase-11, which contributes to activation of a non-canonical NLRP3 inflammasome in response to enteropathogenic bacteria, such as *Citrobacter rodentium* and *Escherichia coli*. Second, they prime the expression of inflammasome-forming NLRP3, RIG-I and AIM2 molecules for potentiating inflammasome activation.

Type I IFNs are innate immune effector molecules with strong pro-inflammatory activities, and have been shown to contribute to the high mortality rates in septic shock syndromes (Karaghiosoff et al., 2003; Huys et al., 2009). Type I IFNs also contribute to inflammasome-dependent caspase-1 activation leading to pro-inflammatory pyroptotic cell death (Figure 2) (Anand et al., 2011; Franchi et al., 2012). There have been multiple different inflammasomes identified that sense a diverse array of microbial- and damage-associated PAMPs. These include the Naip-Nlrc4 inflammasome (Mariathasan et al., 2004; Kofoed and Vance, 2011; Zhao et al., 2011), the Nlrp1b inflammasome (Boyden and Dietrich, 2006; Masters et al., 2012), the Nlrp3 inflammasome (Kanneganti et al., 2006b; Mariathasan et al., 2006; Sutterwala et al., 2006; Anand et al., 2011), the Nlrp6 inflammasome (Elinav et al., 2011), the Nlrp12 inflammasome (Vladimer et al., 2012), the Aim2 inflammasome (Fernandes-Alnemri et al., 2010; Jones et al., 2010; Rathinam et al., 2010; Sauer et al., 2010), the RIG-I inflammasome (Poeck et al., 2010; Pothlichet et al., 2013), and the IFI16 inflammasome (Kerur et al., 2011). Inflammasome-dependent casapase-1 activation and pyroptosis are associated with the production of mature IL-1β and IL-18 cytokines, which generates a pro-inflammatory environment in host tissues (Figure 2). Inflammasome-dependent pyroptosis shares features of both apoptosis and necrosis and is tightly regulated by distinct signaling pathways.

### NLRP3

NLRP3 is the most widely studied inflammasome and it requires two signals for its assembly into an active complex (Kanneganti, 2010; Anand et al., 2011). The first signal is TLR-dependent expression of NLRP3, while the second is often a damage related factor such as production of reactive oxygen species (ROS) or membrane damage (Anand et al., 2011). Activated TLRs transfer signals through two major adapters that specify the downstream signaling pathways (Takeuchi and Akira, 2010). The first one, MyD88 is required for NF-κB activation downstream of all TLRs except TLR3. The second, TRIF plays a dominant role in TLR3-dependent NF-κB activation and TLR4-mediated IRF signaling (Fitzgerald et al., 2003a,b; Sato et al., 2003; Yamamoto et al., 2003).

Recently, our lab and other groups have shown that the TLR4-TRIF axis regulates caspase-11 expression and non-canonical Nlrp3 inflammasome-mediated host defense against enteropathogens, *Escherichia coli* (EHEC), *Citrobacter rodentium*, and *Salmonella Typhimurium* (Kayagaki et al., 2011; Broz et al., 2012; Gurung et al., 2012; Rathinam et al., 2012). Consistent with this, Sander et al. have reported that the gram-negative bacterium *E. coli* induces IFN-β and activates the Nlrp3 inflammasome in the absence of virulence factors other than microbial mRNA (Sander et al., 2011). In addition, Rathinam et al. demonstrated that the IRF3-type I IFN-IFNAR-STAT-1 signaling pathway is indispensable for caspase-11 expression and activation of the Nlrp3 inflammasome and pyroptosis (Rathinam et al., 2012). Furthermore, they demonstrated that IFNβ treatment significantly increased pro-caspase-11 expression and that once induced, caspase-11 undergoes spontaneous activation. Broz et al. have observed that IFNAR-STAT-1 axis is important for caspase-11 activation, but not for its expression in *Salmonella* infected macrophages (Broz et al., 2012). In a subsequent review, they speculated that type I IFN-dependent expression of a yet-unidentified host molecule may trigger caspase-11 activation or that a unknown bacterial signal is required (Broz and Monack, 2013).

Two recent studies have revealed that several gram-negative bacteria, but not gram-positive bacteria, can activate the non-canonical Nlrp3 inflammasome and identified LPS as their common PAMP responsible for the activation of Caspase-11 (Hagar et al., 2013; Kayagaki et al., 2013). Both of these studies have shown that intracellular LPS is sufficient to induce the activation of caspase-11. Furthermore, these studies revealed that a sub-component of LPS, lipid A is sufficient to activate the casaspe-11-dependent non-canonical Nlrp3 inflammasome. They presented evidence that when transfected, intracellular LPS or lipid A could activate the non-canonical Nlrp3 inflammasome independent of TLR4 or the TLR4-associated lipid A-binding proteins MD1 and MD2 and even the TLR4 downstream signaling molecules TRIF and IFNAR receptor molecules. In contrast, a study by Guarda et al. reported that type I IFNs suppress Nlrp1 and Nlrp3 inflammasomes in a STAT1-dependent manner (Guarda et al., 2011). Further studies are required to clarify the role of type I IFNs and their precise mechanisms in the regulation of inflammasome activation. Of particular importance is that the intracellular sensor of lipid A is yet to be identified.

Together, regardless of the exact mechanisms, type I IFNs have clearly emerged as crucial regulators of inflammasome activation and pyroptosis (Figure 2). Physiologically, caspase-11 triggered pyroptosis is required for surveillance against bacteria that enter the cytosol, such as the sifA mutant of *S. Typhimurium*, an sdhA mutant of *Legionella pneumophila* and *Burkholderia* species (Aachoui et al., 2013). However, type I IFNs are known to be exploited by microbial pathogens to induce the death of immune cells and suppress host immune responses. In support of this, caspase-11-mediated cell death is responsible for pathology and detrimental effects *in vivo* (Wang et al., 1998; Kayagaki et al., 2011). For example, Broz et al. showed that wild type *Salmonella* induces caspase-11-mediated cell death in caspase-1-deficient mouse macrophages, and that caspase-11 increases the bacterial virulence and host cell susceptibility to infection (Broz et al., 2012). In addition, *Salmonella* also exploits type I IFN signaling to induce the RIP1-RIP3-dependent necroptotic cell death pathway to kill macrophages (Lindgren et al., 1996; Robinson et al., 2012).

### RIG-I and NLRP3

Viruses are by far the best-known inducers of type I IFNs and have also been recognized to induce activation of distinct inflammasomes, including those comprised of NLRP3, AIM2, and RIG-I. The first evidence of Nlrp3 inflammasome involvement in antiviral responses showed its role in sensing of both viral RNA and its analog poly(I:C) in mouse macrophages (Kanneganti et al., 2006a,b). *In vivo*, Nlrp3 inflammasome activation protected mice from influenza infection (Allen et al., 2009; Thomas et al., 2009). Poeck et al. have reported that Rig-I from mouse macrophages senses cytoplasmic RNA viruses and assembles an inflammasome (Poeck et al., 2010). More recently, RIG-I was reported to induce type I IFN through a MAVS/TRIM25/RNF135 signaling axis following influenza infection, and was shown to have profound effects on NLRP3 inflammasome activation and IL-1β secretion in human lung epithelial cells (Pothlichet et al., 2013). Together, these studies demonstrate that RIG-I can itself assemble an inflammasome and also contributes toward, type I IFN-dependent potentiation of NLRP3 expression (Figure 2).

### AIM2

The cytosolic bacteria *Francisella novicida* and *Listeria* both induce killing of myeloid and lymphoid cells in a manner dependent on Type I IFN signaling, an effect which has been shown to be detrimental to the host (Auerbuch et al., 2004; Carrero et al., 2004; O'Connell et al., 2004; Henry et al., 2007). Several published studies have clearly established that both *Listeria* (Henry et al., 2007; Warren et al., 2008; Kim et al., 2010; Tsuchiya et al., 2010; Wu et al., 2010) and *Francisella* (Henry and Monack, 2007; Fernandes-Alnemri et al., 2010; Jones et al., 2010; Rathinam et al., 2010) activate the Aim2 inflammasome and pyroptosis in mouse macrophages. Type I IFNs prime AIM2 expression (Kotredes and Gamero, 2013) and potentiate cytosolic bacterial DNA recognition for inflammasome activation (Fernandes-Alnemri et al., 2010). Although mice studies show that type I IFN dependent caspase-11 expression is important for the activation of the non-canonical Nlrp3 inflammasome (Kayagaki et al., 2011; Gurung et al., 2012; Rathinam et al., 2012), it is not known if it is also required for AIM2 and RIG-I mediated caspase-1 activation or cell death.

## Conclusions

IFNs were the first cytokines discovered to have immune regulatory capacity. Despite their clinical application in some treatment regimens, we still do not have a complete understanding of the mechanistic effects of IFNs required to further develop treatments that capitalize their full potential therapeutic effects. A vast amount of past research has been focused on the role of IFNs as anti-viral molecules with a limited number of studies for other microbial infections. Recent studies have clearly indicated a dual role for type I IFNs both in infectious and inflammatory diseases. Despite the potential benefits, it is often challenging to manipulate type I IFNs for therapeutic purposes due to their role in regulating the expression and activation of a huge number of downstream genes, often complicating the conclusions. Part of the problem is that types I IFNs exert differential immunomodulation on diverse cell types, environments, and varying physiological conditions. The pathogen- and host-mediated counter regulatory pathways further complicate IFN-induced responses in cell death and disease.

Recently, type I IFNs were recognized as crucial regulators of non-canonical NLRP3 inflammasome activation and pyroptosis. Although the majority of literature indicates a positive role for inflammasomes in anti-microbial host defense, recent reports indicate detrimental effects due to excessive cell death, inflammation, and collateral tissue damage in vital organs (Lupfer and Kanneganti, 2012). A paradox exists, where inflammasomes intended to defend against cytoplasmic invaders by pyroptosis, which is predominantly protective *in vitro*, however in *in vivo*, if exceeds a certain level, can lead to cell and tissue damage and organ failure resulting in negative outcomes. The paradox for type-I IFNs is that their ability to inhibit microbial spread by inducing cell death is counteracted by apoptotic depletion of immune cells and inhibiting anti-microbial immune responses leading to immune suppression. Future research should explore the detailed molecular mechanisms that are responsible for type I IFN-dependent cell death and inflammasomes activation in the context of immunity and immunopathology. These findings may lead to better-targeted therapeutic interventions to treat inflammatory and infectious diseases.

### Conflict of interest statement

The authors declare that the research was conducted in the absence of any commercial or financial relationships that could be construed as a potential conflict of interest.

## Abbreviations

IFN-I: type I interferon
IFNAR: Interferon-α/β receptor
ISRE: Interferon stimulated response element
ISGs: Interferon stimulated genes
TLR: TOLL-like receptor
NLR: NOD-like receptor
